# The synergistic interference effect of silica nanoparticles concentration and the wavelength of ELISA on the colorimetric assay of cell toxicity

**DOI:** 10.1038/s41598-021-92419-1

**Published:** 2021-07-23

**Authors:** Fariba Abbasi, Hassan Hashemi, Mohammad Reza Samaei, Amir SavarDashtaki, Abooalfazl Azhdarpoor, Mohammad Javad Fallahi

**Affiliations:** 1 Department of Environmental Health Engineering, School of Health, Shiraz University of Medical Sciences, Shiraz, Iran; 2 Department of Medical Biotechnology, School of Advanced Medical Sciences and Technologies, Shiraz University of Medical Sciences, Shiraz, Iran; 3 Department of Internal Medicine, School of Medicine, Shiraz University of Medical Sciences, Shiraz, Iran

**Keywords:** Biological techniques, Cell biology

## Abstract

The 3-(4,5-dimethylthiazol-2-yl)-2,5-diphenyltetrazolium bromide (MTT) assay is the most common method for the determination of cell toxicity, but some factors limit the sensitivity of this method, such as pH. Less attention had been paid to the interference effect of optical and plasmonic properties of SiO_2_ nanoparticles (NPs) in the wavelength range assigned to MTT. This study investigated the synergistic interference effect of SiO_2_ NPs and wavelength on MTT assay for the first time. The examined variables included the type of SiO_2_ NPs concentrations (1, 10, and 100 mM) and different wavelengths (470, 490, 520, and 570 nm). The results showed that optical density (OD) increased (p < 0.05) when wavelength and the concentration of crystalline SiO_2_ NPs increased. So, the maximum OD at 10 and 100 mM were attributed to crystalline SiO_2_ NPs (p < 0.05) due to the functional group, whereas it was related to amorphous at 1 mM (p > 0.05). According to polynomial regression modeling (PRM), the maximum interference effect was predicted at crystalline SiO_2_ NPs and wavelength > 550 nm. Besides, the synergistic effects of SiO_2_ NPs, wavelength, and concentration of NPs had been a good fitting with first-order PRM. Thus, the concentration of SiO_2_ NPs had a confounder factor in colorimetric for MTT assay. The best artificial neural network (ANN) structure was related to the 3:7:1 network (R_all_ = 0.936, MSE = 0.0006, MAPE = 0.063). The correlation between the actual and predicted data was 0.88. As SiO_2_ NPs presence is an interfering factor in MTT assay concerning wavelength, it is suggested wavelength use with minimum confounding effect for MTT assay.

## Introduction

3-(4,5-Dimethylthiazol-2-yl)-2,5-diphenyltetrazolium bromide (MTT) is one of the most common methods for the determination of cell proliferation and cellular toxicity assay. MTT is an attractive method compare to others, such as methylene blue^1,2^. It is widely used to determine the toxicity effects of various drugs and nanoparticles in industry and medicine. This method is based on the colorimetric assay, that tetrazolium salt is reduced to formazan by donor electron. This reaction can be due to the presence of Nicotinamide adenine dinucleotide (NADH) produced by the mitochondria of living cells, as well as superoxide^3–6^. The approximate level of living cells is estimated based on the purple dye intensity^7^. Although this is a simple and repeatable method^8^, it has limitations such as nonspecific adsorption, relatively poor sensitivity, and low surface to volume ratio. These limitations in some cases can challenge, and invalidate the evaluation process basis^9^. Previous studies have shown that certain conditions, such as antioxidants, change of pH range, reducing agents, temperature variation, and the type of solvents, are the disruption factors on regeneration formazan and MTT test^6,10–12^. On the other hand, some studies have suggested a reaction between nanoparticles and these efficient factors in MTT. In Popescu’s study, the MTT-formazan complex was formed on TiO_2_ NPs^13^. In this condition, about 14% of false viability in the MTT was related to the TiO2-MTT reaction^14^. Moreover, Fe^3+^ are acted as a strong reaction inhibitor that is interfered with the MTT assay^13^. Other structures, such as graphene, be efficient in light-absorbing and transmitting during the colorimetric process^15^. Thus, the same effects may be due to the presence of other substances and NPs. SiO_2_ nanoparticles (NPs) are stable NPs^16^, that are widely used in industry, medicine, and pharmaceuticals. Besides, its crystalline structures are an environmental pollutant that can oxidize the human organs^17^. Commonly MTT assay is used to determine the effectiveness or toxicity of SiO_2_ NPs on invitro. However, there has been less attention paid to the interference effects of SiO_2_ NPs on MTT qualitatively and quantitatively because they have been introduced as a surface reactiveness agent that can produce radicals such as OH^0^. Therefore, it can involve in redox reaction and amplify the tetrazolium reduction^18,19^. Moreover, formazan is a pH-sensitive dye that also binds to the surface of SiO_2_ NPs^20^. Finally, the silica matrix is encapsulated during the polymerization process of color molecules^21^. On the other hand, silica has certain optical properties that can affect colorimetric assay, as light transmission and reflection^22^. These differences and properties can be changed during the different wavelengths that become more significant with nanoparticle crystallization. The amounts of light transmission from silica have gradually increased at a wavelength < 500 nm, whereas the maximum absorption was at 520 nm^23–25^. According to logarithmic slope in light transmission and the maximum capability of SiO_2_ NPs for adsorption in 400–600 nm range, as maximum adsorption of formazan at 570 nm^6^. It is expected that the co-presence of silica and formazan can disrupt the actual results of MTT because MTT is evaluated in the same wavelength range. These interference effects have been less studied, and the relationships between them properly have not been defined. Most events in vitro and real scale have nonlinear relationships that require the use of nonlinear models. Due to this necessity and with the hypothesis of the interfering factor of these nanoparticles, it is necessary to study the nonlinear relationship to predict the MTT reading rate by ELISA reader. Among nonlinear methods, the artificial neural network (ANN) is more accurate in predicts of events. Besides, MTT is based on colorimetric assay and optical properties attributed to the wavelength performance^26^. Moreover, the optical and plasmonic properties of silica resonance increase in the wavelength range of MTT as increasing light absorption with time increases^27,28^. This study aimed to investigate the interference effect of SiO_2_ NPs, their concentrations on MTT assay, and its modeling using regression and ANN model.

## Materials and methods

### Synthesis of SiO_2_ nano particles

In this study, SiO_2_ NPs were synthesized using the Stober method^29^. This method is one of the well-known sol–gel methods for synthesizing nanoparticles. According to this method, Tetraethyl orthosilicate (TEOS, Merck Germany ≥ 99.0%) was used as the raw material for SiO_2_ NPs synthesis. Ethanol (Merck Germany 100.0%) and ammonia (Merck Germany 25.0%) were used as solvent and catalyst, respectively. Deionized water (Merck Germany) was also used for dilution. The synthesis was performed in an ultrasonic bath, after aging, the obtained gel was washed with ethanol and water. Then the product was dried at 24 °C for 24 h. Nanoparticles were also crystallized using thermal methods at different temperatures (350, 600, 800, and 1000 °C). X-ray powder diffraction (XRD) graph was used to investigate the crystallization degree of NPs. Then field emission scanning electron microscopy (FESEM) and energy dispersive spectroscopy (EDS) results were interpreted to determine the size and purity percentage of NPs. Also, NPs distribution in the solution was determined by dynamic light scattering (DLS) ^30^.

### Experiment design

This study was a full factorial design. The investigated variables was included the wavelength used for the enzyme-linked immunosorbent assay (ELISA) reader, the calcination temperature (CT) of the NPs, and the concentration of NPs in the cell culture media. The total number of experimental conditions were assessed was 60 with triplicate. The range of all variables examined is shown in Table 1.

**Table 1:** The investigated variables.

Variables	Unite	The range of variables
Wavelength	nm	470, 490, 520, and 570
Calcination temperature	°C	70, 350, 600, 800 and 1000
Concentration of SiO_2_ NPs	mM	1, 10, and 100

### Experimental analysis

To determine the SiO_2_ NPs interference effect on the MTT assay, the SiO_2_ NPs solutions were prepared at 1, 10, and 100 mM. Later they were transferred into media containing MTT salt. Therefore, MTT assay conditions were simulated, and the prepared solution was incubated at 37 °C for four hr. Finally, the purple color obtained from the MTT assay was read by the ELISA. For determination of the wavelength effect, optical density (OD) was determined at 470, 490, 520, and 570 nm.

### Artificial neural network modelling

ANN is one of the modeling methods to determine the nonlinear relationship between variables which has several input variables, hidden, and output layers. In this model, the number of neurons in each hidden layer has a significant effect on the response. In the present study, using MATLAB 2018, the feed-forward backpropagation algorithm (Levernberg–Marquardt algorithm) was used for modeling. Initially, the network was trained with OD obtained at different wavelengths (470, 490, 520, and 570 nm) obtained from the ELISA. In this model, 70, 15, and 15% of the data were used for training, validation, and testing, respectively. After training, the amount of error validation was monitored, and after increasing the error with a specific repetition, training stops. The best ANN structure was determined based on mean square error (MSE), mean absolute percentage error (MAPE), and correlation coefficient (R) in the hidden layer, which was used to predict the interfering effect of SiO_2_ NPs on OD obtained from MTT assay. A hidden layer is located between the input and output of the algorithm. It is applied for weighting to inputs to provide the output. The performance of the hidden layer base on nonlinear function and the efficiency of ANN associated with the layer significantly. In this study, the number of neurons in the hidden layer was determined based on the most comprehensive equation in environmental toxicology, such as nanoparticles toxicology. It is expressed in both Eqs. (1) and (2):

1 $$ \frac{{2\left( {i + o} \right)}}{3} < n < i\left( {i + o} \right) - 1, $$

2 $$ 0.{\text{5i}} - {\text{2}} < {\text{n}} < {\text{2i}} + {\text{2,}} $$

i is the number of inputs, o is the the number of outputs and n is the number of hidden layer neuron.

### Statistical analysis

OD at 470, 490, 520, and 570 nm were determined using descriptive statistics. It was compared at each stage using a one-way analysis of variance (ANOVA) test at different temperatures. The correlation between OD and the variables was performed by Spearman correlation. The significant difference was adjusted to 0.05. The interference effect of nanoparticles Modeling was performed using linear, cubic, quadric, surface response, and polynomial regression models. Then the best regression model was selected based on R^2^, adjusted-R^2^, MAPE, and root-mean-square error (RMSE). Statistical analysis, tests, and modeling have been performed in MATLAB 2018.

### Ethics approval and consent to participate

All ethical aspects of this study were approved by Shiraz University of medical science’ Ethics Committee (IR.SUMS.REC.1398.1226).

### Consent for publication

Not applicable.

## Results and discussion

### The characteristic of SiO_2_ NPs

**Figure 1:**
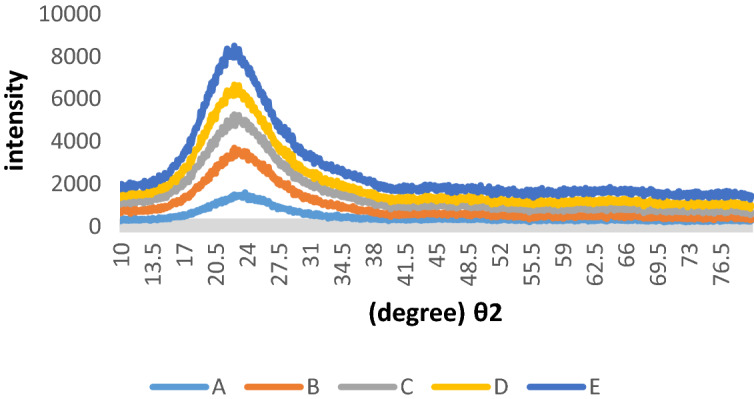
XRD patterns of SiO_2_ NPs (A) dried at 70 °C, (B) calcinated at 350 °C, (C) calcinated at 600 °C, (D) calcinated at 800 °C, and (E) calcinated at 1000 °C.

In this study, the crystallization process of SiO_2_ NPs was investigated during CT between 70 and 1000 °C, as shown in Fig. 1.

The XRD peak in all nanoparticles was 22–24 °C (Fig. 1). They were confirmed that the SiO_2_ NPs were converted to crystal lattices during the increase of CT. In previous studies, the XRD peak for SiO_2_ NPs in amorphous and crystal was 14°–21° and 26°–27°, respectively^31–33^. Besides, crystallization increased with increasing calcination temperature up to 1000 °C because amorphous SiO_2_ NPs were formed at low temperatures (70–150 °C)^34^. Instead, as crystallization increases (from A to E), regular lattices were created. Subsequently, the NPs surface reactivity was reduced, and strong bonds were formed between silica and oxygen.

SiO_2_ NPs have been identified in the range of 400–1400 cm^−1^ in the Fourier-transform infrared spectroscopy (FTIR) spectra^35^. Some of the functional groups in FTIR spectra of investigated SiO_2_ NPs are expressed in Table 2.

**Table 2:** The functional group of SiO_2_ NPs (A) amorphous, (B) calcinated at 350 °C, (C) calcinated at 600 °C, (D) calcinated at 800 °C, and (E) calcinated at 1000 °C.

SiO_2_ NPs	Bonds
A	Si–O, Si–OH, Si–O–Si, Si–Cl
B	Si–O, Si–OH, Si–O–Si, Si–Cl, O–H
C	Si–O, Si–O–Si, Si–Cl, Si–H
D	Si–O, Si–OH, Si–O–Si, Si–Cl, O–H, hydroxyl group, ≡Si–O–Si≡
E	Si–O, Si–OH, Si–O–Si, Si–Cl, O–H, hydroxyl group, ≡Si–O–Si≡

According to Table 2, the maximum light transmittance in all nanoparticles was in the range of 1070–1085 cm^−1^. This range was determined the bond between Si–O and the functional groups coupled to this bond^36–38^. So, the presence of the Si–OCH_3_ bond was confirmed in E NPs. This bond is an active functional group that reacts to more substance. In addition, there was a spectrum between 616 and 797 cm^−1^ in D and E NPs that identified as depicted siloxane. The triple bond in depicted siloxane is an effective factor in the reactivity of SiO_2_ NPs^39^. Thus, increasing the calcination temperature increased the crystallinity of SiO_2_ NPs, but more investigation was essential to determine the regeneration of formazan in MTT assay and its interference-effect.

### Interference effect of SiO_2_ NPs on OD during the CT at different wavelengths

**Figure 2:**
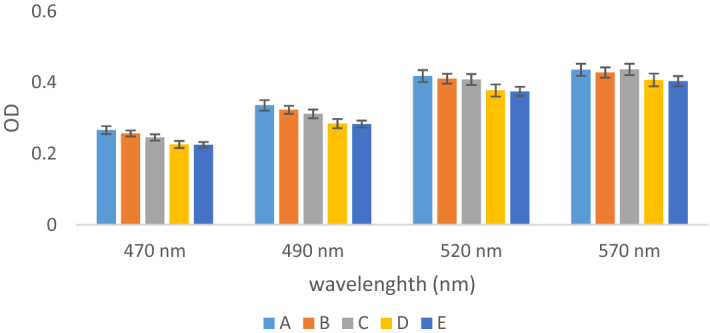
The ratio of OD of SiO_2_ NPs compare to control sample during different wavelength (A) amorphous, (B) calcinated at 350 °C, (C) calcinated at 600 °C, (D) calcinated at 800 °C, and (E) calcinated at 1000 °C.

The OD percent due to SiO_2_ NPs (A, B, C, D, and E) at four wavelengths of 470, 490, 520, and 570 nm is shown in Fig. 2.

**Figure 3:**
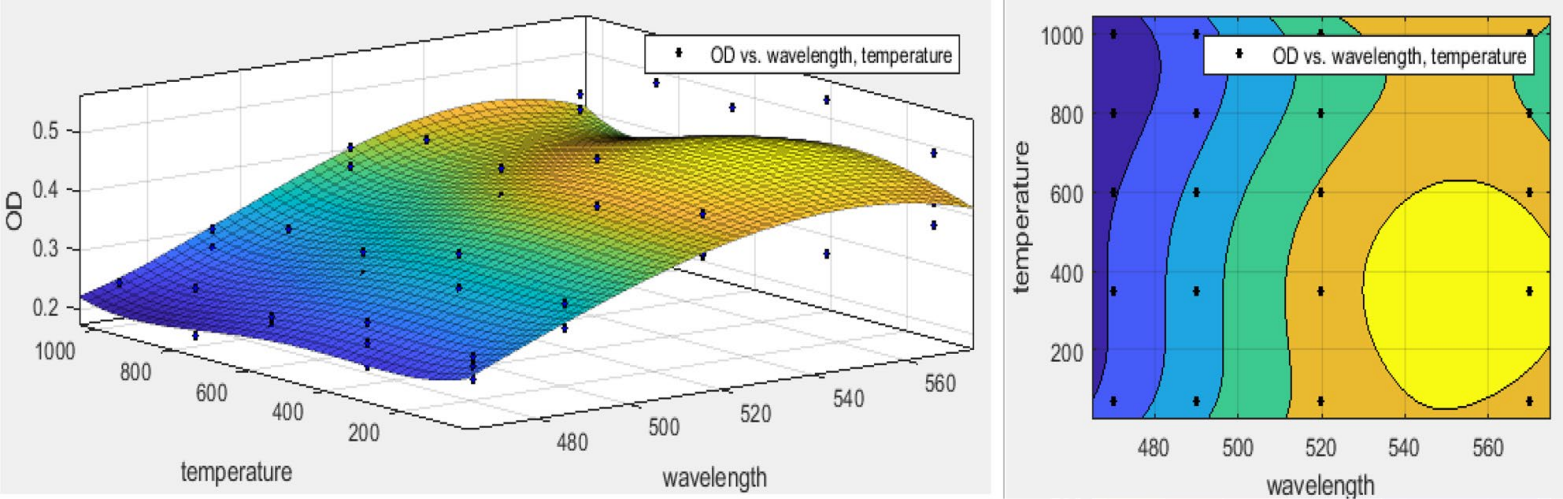
The synergistic effect of wavelength and calcination temperature of SiO_2_ NPs using polynomial regression model.

According to Fig. 2, the OD was increased as wavelengths increasing. So, maximum OD was determined at the wavelength of 570 nm (p < 0.0001) because the refractive index decreases with increasing wavelength. This index showed the speed of light passing through a substance^40^. Light transmission and maximum absorption amount significantly were dependent on the alloy crystal and its size^23^. It was reported that wavelength increasing in 400–600 nm, the amount of light transmission increased from 20 to 70%^22^. Despite it was increased exponentially and was became almost constant after 600 nm^41^. In this study, the OD was decreased when the calcination temperature was increased in all wavelengths (p < 0.005). The results of Matysiak et al. study showed that during the crystallization of SiO_2_ NPs, the absorption increased at a wavelength > 325 nm. Consequently, the light transmittance decreased because the content of Si increased in crystal nanoparticles^26^. Polynomial regression modeling showed that the maximum interference effect of wavelength and calcination temperature of SiO_2_ NPs had been related to the temperature range of in the range of 70–600 °C as well as wavelength 550 nm (Fig. 3). The equation of this relationship is expressed in Eq. (3).

3 $$ \begin{aligned} {\text{f}}\left( {{\text{x}},{\text{y}}} \right){\text{ }} & = {\text{ 16}}.{\text{18 }}{-}{\text{ }}0.{\text{1x }} + {\text{ }}0.0{\text{3y }} + {\text{ }}0.000{\text{2x}}^{{\text{2}}} {-}{\text{ }}0.000{\text{2xy }} \\  & \quad + {\text{ }}0.00000{\text{6y}}^{{\text{2}}} {-}{\text{ }}0.000000{\text{2x}}^{{\text{3}}} + {\text{ }}0.000000{\text{3x}}^{{\text{2}}} {\text{y }}{-}{\text{8}} \times {\text{1}}0^{{ - {\text{9}}}} {\text{xy}}^{{\text{2}}} \\  & \quad {-}{\text{4}}.{\text{6}} \times {\text{1}}0^{{ - {\text{9}}}} {\text{y}}^{{\text{3}}} {-}{\text{2}}.{\text{3}} \times {\text{1}}0^{{ - {\text{1}}0}} {\text{x}}^{{\text{3}}} {\text{y}}{-}{\text{1}}.{\text{9}} \times {\text{1}}0^{{ - {\text{12}}}} {\text{x}}^{{\text{2}}} {\text{y}}^{{\text{2}}} + {\text{5}}.{\text{8}} \times {\text{1}}0^{{ - {\text{12}}}} {\text{xy}}^{{\text{3}}} + {\text{9}}.{\text{1}} \times {\text{1}}0^{{ - {\text{13}}}} {\text{y}}^{{\text{4}}} . \\ \end{aligned} $$

That

X is the wavelength (nm) and y is the temperature (°C).

According to Eq. (3) and Table 3, R-square > 0.9 and MAPE < 10%. Moreover, there was no difference between R-squared and adjusted R-square. Therefore, all of them confirmed that the cubic polynomial regression model had excellent forecasting for the prediction of the interference effect of wavelength and temperature on OD in MTT assay^42^.

**Table 3:** The performance of polynomial regression model for prediction of interference effect of variables.

The interference of parameters	SSE	R-square	Adjusted R-square	RMSE	Effective weight	MAPE	Fitted model
Concentration and temperature	0.61	0.94	0.94	0.1	Non	0.4	Cubic
Wavelength and temperature	0.1375	0.7	0.62	0.05	Concentration	0.1	Linear
Concentration and wavelength	0.037	0.921	0.9137	0.02606	Non	0.48	Quadratic

According to Fig. 3, although the light can pass from the media containing SiO_2_ NPs at higher wavelengths^43–45^ and were functional groups on D and E NPs (Table 2). It may involve in oxidation–reduction reaction and even bonded to formazan. Hence, the presence of SiO_2_ NPs was a confounder factor in the colorimetric of MTT assay, especially during higher wavelengths (Fig. 3). Based on these results, it is best to use an MTT assay for the determination of crystalline nanoparticles toxicity that was calcinated at high temperatures. It was suggested that the wavelengths < 500 nm used to determine the toxicity of SiO_2_ NPs.

### The effect of SiO_2_ NPs concentration on OD

**Figure 4:**
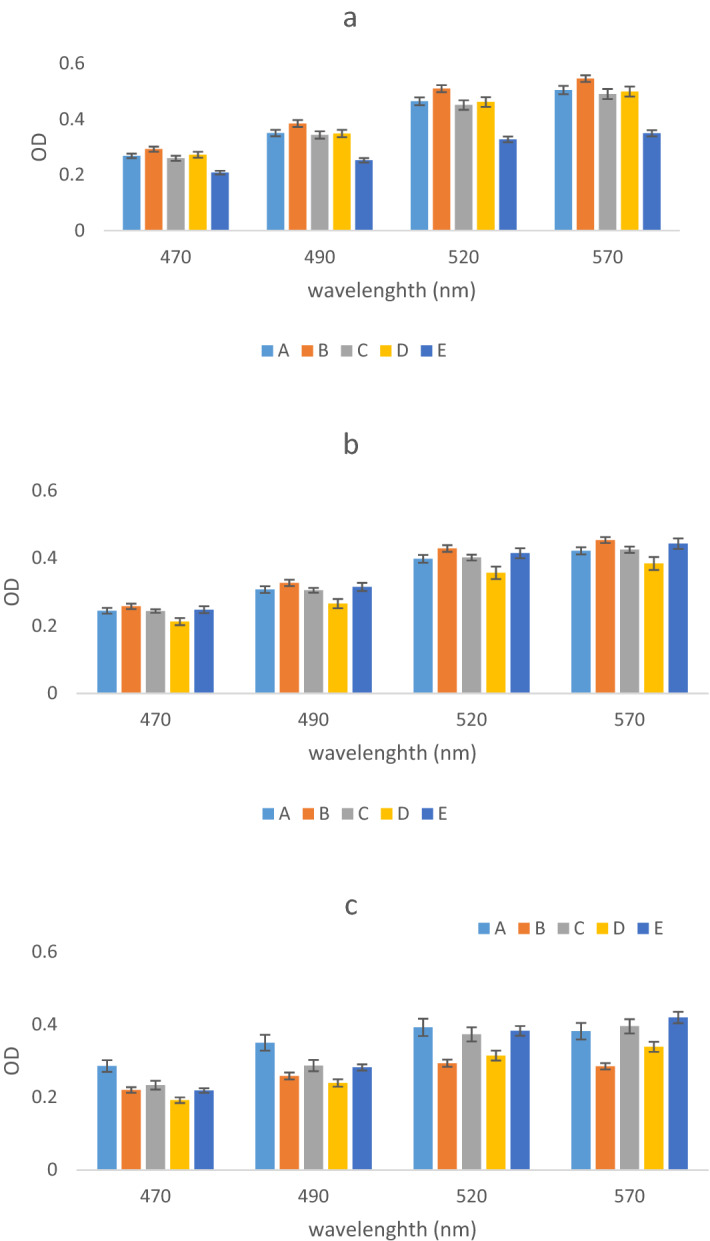
The OD ration of SiO_2_ NPs compare to control sample during different wavelength (**a**) 1 mM, (**b**) 10 mM and (**c**) 100 mM. (A) amorphous, (B) calcinated at 350 °C, (C) calcinated at 600 °C, (D) calcinated at 800 °C, and (E) calcinated at 1000 °C.

The OD attributed to concentrations of 1, 10, and 100 mM of SiO_2_ NPs are shown in Fig. 4.

As shown in the 1 mM concentration, OD increased with increasing wavelength (Fig. 4a). The OD of A, B, C, D, and E were detected between 0.27–0.5 (p < 0.0001), 0.29–0.54, 0.26–0.49, 0.19–0.33, and 0.27–0.499. Hence, the highest coefficient of variation (CV) was related to all concentrations of B NPs (p < 0.0001), but with increasing calcination temperature, CV has decreased (p < 0.05). Nevertheless, the maximum and minimum ODs were determined in the media containing B and D NPs (p < 0.05) which, were calcinated at 350 °C and 600 °C, respectively.

According to Fig. 4b (concentrations = 10 mM), OD level of A, B, C, D and E NPs were 0.24–0.42 (p > 0.05), 0.257–0.45, 0.23–0.39, 0.21–0.38, and 0.247–0.443 (p < 0.05), respectively. OD was increased during the increasing of wavelengths (p < 0.05). At all studied wavelengths, there was an insignificance difference between the minimum and maximum OD in E and B NPs (p > 0.05).

**Figure 5:**
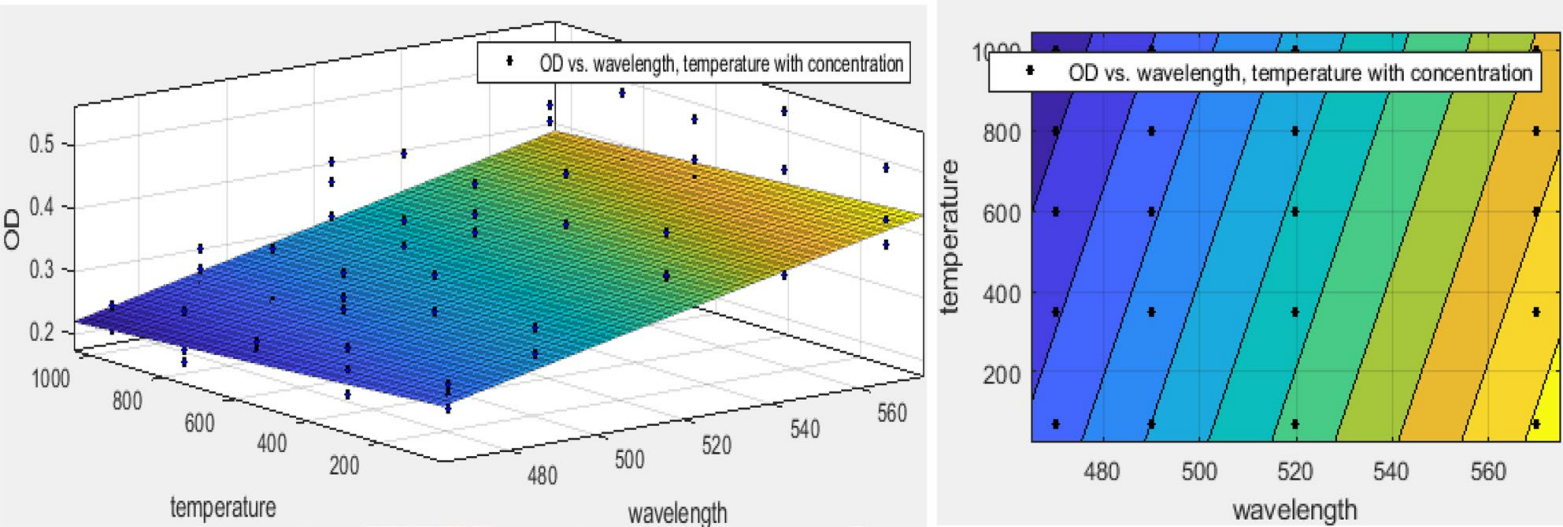
The synergistic effect of wavelength, calcination temperature and concentration of SiO_2_ NPs using polynomial regression model.

At a concentration = 100 mM, by wavelength increasing, the OD has increased (Fig. 4c). The OD of A, B, C, D, and E were 0.286–0.382 (p < 0.0001), 0.22–0.29, 0.22–0.29, 0.21–0.34, and 0.22–0.42 (p < 0.0001), respectively. The maximum and minimum ODs were attributed to E and D NPs (p < 0.05), respectively. Also, the highest CV was estimated in E NPs (p < 0.0001). The Pearson test showed an insignificant correlation between CT and OD (p < 0.05). In low concentrations, increasing calcination temperature be effective in increasing OD (p < 0.04). While, in high concentrations, a similar trend has not been observed (p > 0.05). Also, concerning the effect of NPs concentration, the polynomial regression models have been shown a significant effect related to the simultaneous influence of CT and wavelength with 95% confidence bounds (R-square: 0.94 and RMSE: 0.1) as well as the weighting with the concentration of SiO_2_ NPs. Therefore, the weighting of this model with concentration was predicted well by PRM (first-order equation). although R-square > 0.6 shown that it might be satisfactory in some applications, the insignificant difference between R-squared and adjusted R-square (< 0.01) as MAPE = 10 confirmed the capability of this model (Table 3)^42^. The equation of this relationship and its three-dimensional diagram are shown in Eq. (4) and Fig. 5, respectively.

4 $$ {\text{f}}\left( {{\text{x}},{\text{y}}} \right){\text{ }} = {\text{ }}0.00{\text{15x }}{-}{\text{ }}0.0000{\text{4y }} - {\text{ }}0.{\text{4428,}} $$

X is the wavelength (nm) and y is the calcination temperature (°C).

**Figure 6:**
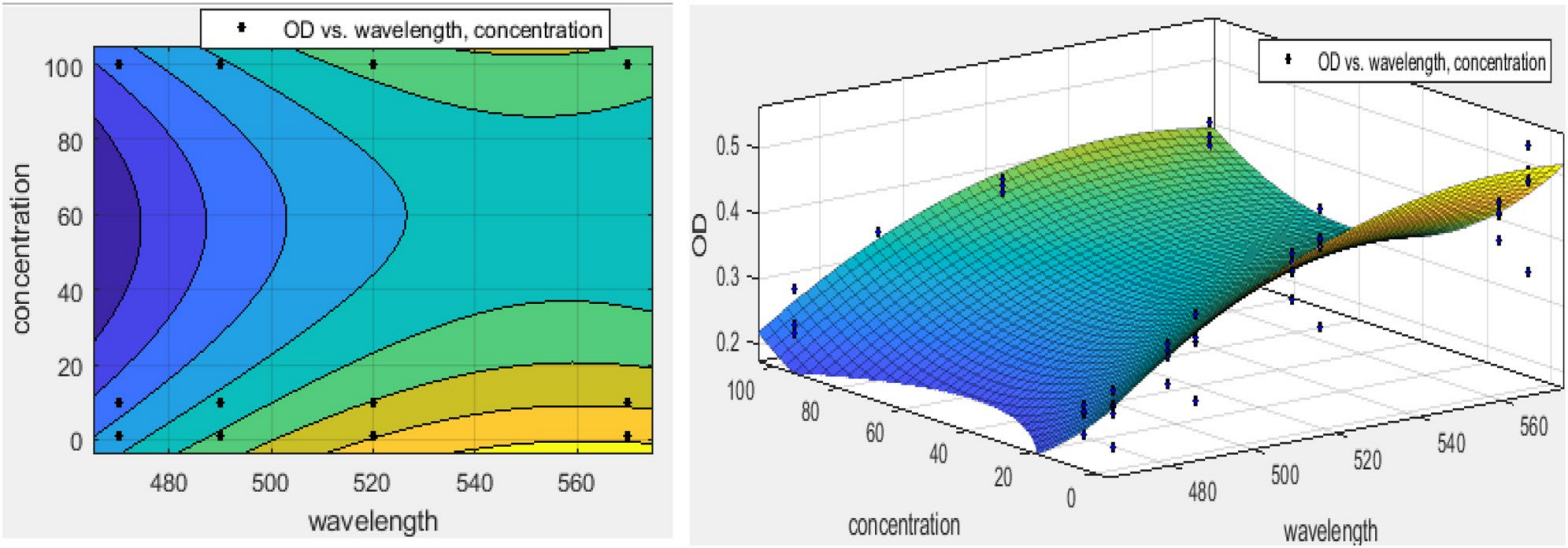
The synergistic effect of wavelength and concentration of SiO_2_ NPs using polynomial regression model.

Based on Fig. 6, there was no significant difference between OD of 1, 10, and 100 mM (p > 0.05). With the increasing wavelength of the ELISA, significant differences were observed (p < 0.05). The regression model was shown the synergistic effects of all variables of concentration, wavelength, and incubation temperature. Remarkably, it was found that the concentration of silica nanoparticles was a confounder factor in colorimetric used in MTT assay because the concentration of silica has been identified as an interfering factor in the absorption and transmission of light^26^. The maximum interference effect of SiO_2_ NPs was at wavelengths = 560 nm and in amorphous nanoparticles (Fig. 5). This result was increased with increasing the SiO_2_ NPs concentration. Each of these variables can be attributed to another as the calcination temperature of SiO_2_ NPs and the ELISA wavelength. Therefore, the simultaneous effect of the wavelength, concentration, and calcination temperature investigated by regression models. The regression modeling of simultaneous effects of wavelength and NPs concentrations using the surface response is shown in Fig. 6.

Moreover, the equation of this interaction was expressed in Eq. (5).

5 $$ {\text{f}}\left( {{\text{x}},{\text{ y}}} \right){\text{ }} = {\text{ }}0.0{\text{3x }} - {\text{ }}0.00{\text{26y }} - {\text{ }}0.0000{\text{2x}}^{{\text{2}}} - {\text{ }}0.00000{\text{5xy }} + {\text{ }}0.0000{\text{46y}}^{{\text{2}}} - {\text{ 7}}.{\text{73,}} $$

X is the wavelength (nm) and y is the concentration of SiO_2_ NPs (mM).

As shown in Fig. 6, the interfering effects of SiO_2_ NPs were increased at concentrations lesser and higher than 20 mM 100 mM, respectively. Moreover, the peak of interference effect was predicted at lower concentrations (R-square: 0.921, MAPE = 0.48, and RMSE: 0.02606) without a variables selection as a weight of the model. Concerning Table 3, R-square > 0.9, the insignificant difference between R-squared and adjusted R-square (< 0.01) as well as MAPE = 20–50% (MAPE = 48%). This model had good forecasting for the prediction of this interference effect^42^. No such interference effects have been observed during wavelength < 480 nm. It can be concluded that the selection of wavelength for MTT assay has been related to the concentration of SiO_2_ NPs.

### The modelling of interference of SiO_2_ on OD using ANN

**Figure 7:**
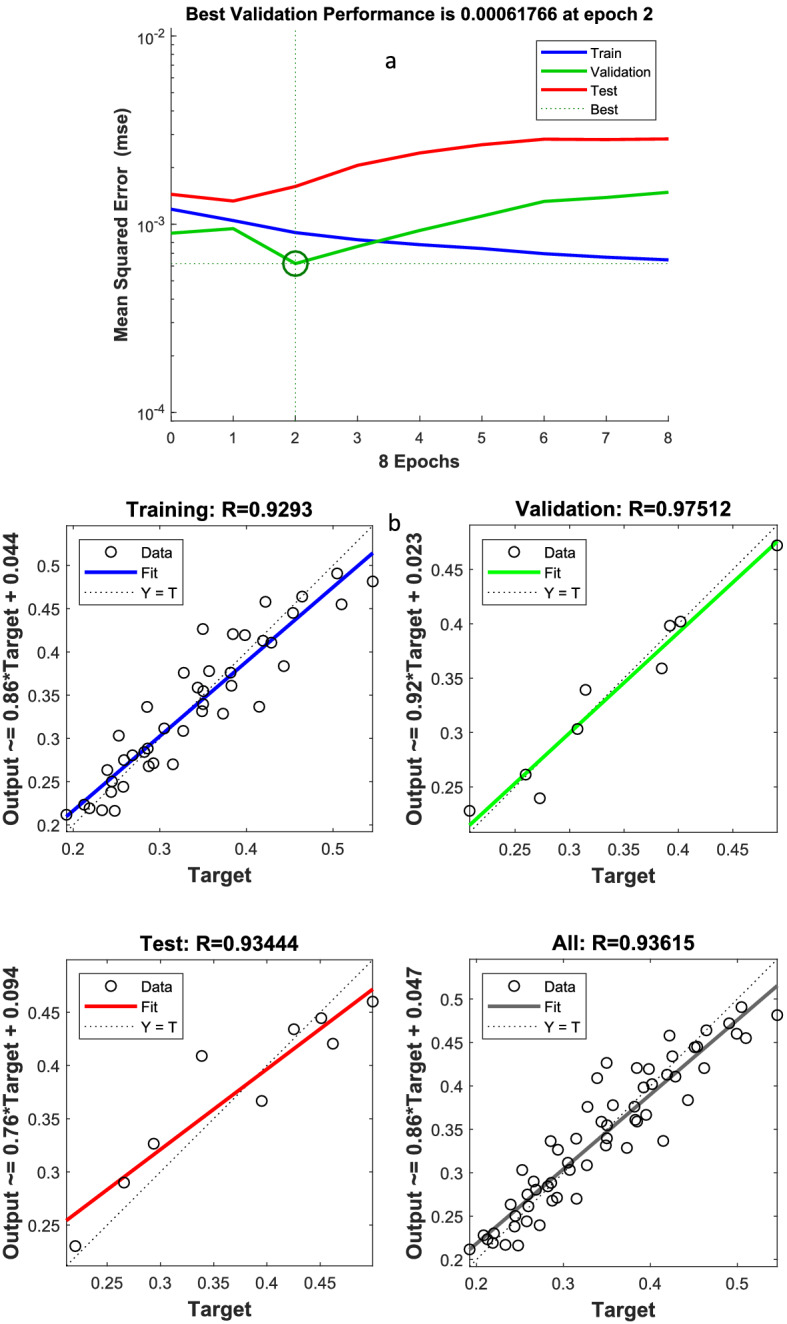
The MSE and regression of the best model for prediction of wavelength transmission.

According to Fig. 3, the simultaneous effect of CT and wavelength has been fittied with polynomial regression models (R-square: 0.7 and RMSE: 0.05). The R^2^ > 0.6 may be satisfactory in some applications therefore, the regression model couldn’t be the most effective model for the prediction of some relationship between both parameters. Other studies used machine learning methods to predict cell toxicity as deep learning, random forests, k-nearest neighbors, and support vector machines^46^. But the use of ANN has been introduced as a useful, reliable, cheap, and fast tool for predicting the non-linear relationship between variables such as OD in MTT^47^. The Levernberg–Marquardt (LM) algorithm was used to determine the best network structure for weight and bias. Because these algorithms have the a higher predictive capacity for the toxicity of nanoparticles in biology media compare to others as deep neural networks^48,49^. According to this method, the number of neurons the in hidden layer based on Eqs. (1) and (2) were 2.6 to 11 and − 0.5 to 8, respectively. Then the intersection of both intervals (3–8 neurons) was selected for ANN training. Based on the number of input and output variables, the number of neurons in the hidden layer = 3–8 was used to predict wavelength transmission using ANN. The best of them according to Table 4 was related to 7 neurons in the hidden layer. After all, it had the highest correlation coefficient (R_all_ = 0.936, R_training_ = 0.929, R_validation_ = 0.975 and R_test_ = 0.934) and the lowest MSE = 0.0006 as well as MAPE = 0.063 because the MAPE is less sensitive to outliers. Moreover, the gradient of this structure was lower than others (Gradient = 0.0001). On another word, the prediction of ANN was more than PRM (see Tables 3 and 4). Besides, Fig. 7 also shows the structure of the best predictive model and the relationship between input and output by the hidden layer. Because the trend of validation and test error was similar, and there was no overfitting before the epoch of 2. But the validation error increased for seven iterations significantly that training is stopped based on the stop algorithm. Moreover, the regression coefficient in Fig. 7b showed the correlation in the test dataset = 0.934 and all test = 0.937.

**Table 4:** The results of the number of neurons in hidden layer (4–8 neuron).

Structure of ANN	R (all)	R (test)	R (training)	R (validation)	MSE	MAPE	Optimum epochs	Stop validation	Gradient range
3:8:1	0.902	0.95	0.889	0.918	0.0011	0.074	4	10	10^–2^–10^–4^ (0.0008)
3:7:1	0.936	0.934	0.929	0.975	0.0006	0.063	2	8	10^–3^–10^–4^ (0.0001)
3:6:1	0.932	0.958	0.928	0.957	0.00099	0.071	8	14	10^–2^–10^–4^ (0.0007)
3:5:1	0.923	0.956	0.914	0.965	0.0013	0.086	1	6	10^–2^–10^–4^ (0.0004)
3:4:1	0.908	0.877	0.920	0.802	0.002	0.23	5	11	10^–2^–10^–4^ (0.0003)

**Figure 8:**
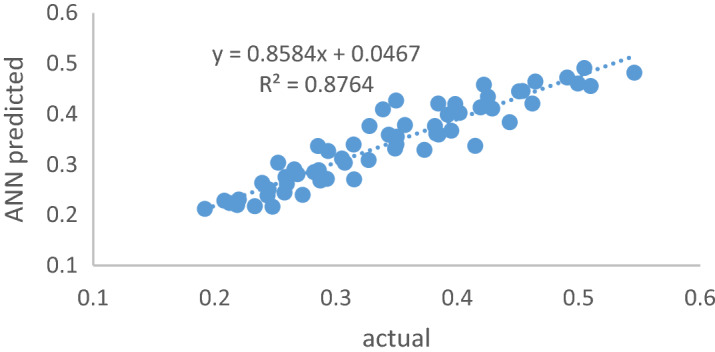
The wavelength transmission for the actual laboratory results and the predicted by ANN model.

The prediction using ANN compared to the experimental value is shown in Table S1. The maximum and minimum transmission wavelengths in real samples were 1 mM and 100 mM, respectively, which were 350 °C and 800 °C (runs of 16 and 45). In the ANN results, the minimum light transmission at a concentration of 100 mM was 470 nm and was related to calcinated nanoparticles at a temperature of 800 °C. The maximum prediction results by ANN were for amorphous nanoparticles, 1 mM concentration, and 570 nm wavelength, respectively. Thus, the neural network well can predict the interference effect of nanoparticles on OD. It has a limit to predict the amount of OD at higher wavelengths. In Fig. 8, the correlation between the values predicted by the ANN model versus the actual value obtained from the laboratory data was 0.88, which shows an acceptable correlation between the network output and the transmission wavelength. Therefore, the ANN had a suitable ability to predict the wavelength transmission of media containing different amorphous types and crystalline nanoparticles with different concentrations. But in addition to these results, further studies on light absorption and reflection and its prediction using other nonlinear models are suggested.

### Sensitivity analysis

In this study, the effect of some parameters on MTT assay was investigated and modeled by ANN. But the determination of the most effective of these parameters is an essential step to designing further studies. Hence, identifying and focusing on effective parameters can decrease the actual interference and errors in the laboratory test. Therefore, we use the sensitivity analysis that corresponds to statistical techniques focused on determining how the variations of the M input variables of a mathematical model influence the response value^50^. According to enter linear regression model, the beta coefficient was used to predict the effect of the studied variables. This correlation was 0.038, − 0.429, and − 0.241 for calcination temperature of silica nanoparticles, nanoparticle concentration, and the wavelength of ELISA-reader, respectively. Among the studied variables, the effective factor was nanoparticles concentration, which was negatively related to OD amount. This correlation was less for decreasing wavelength and increasing the calcination temperature of nanoparticles. Consequently, the calcination of nanoparticles was the most important interference effect on OD of ELISA-reader.

## Conclusion and suggestion

This study investigated the interference effect of different concentrations of SiO_2_ NPs on MTT assay during different wavelengths and modeled by ANN. According to results, the crystallization rate of SiO_2_ NPs and the existence of reactive functional group as Si–OCH_3_ and depicted siloxane bonds has increased with increasing CT up to 1000 °C. The OD level increased with wavelength increasing and decreased the CT. So, the maximum OD was determined at the wavelength = 570 nm (p < 0.0001). Based on PRM, the maximum interference effect of wavelength and CT of NPs were attributed to the temperature range of 70–600 °C and wavelength 550 nm. Moreover, OD was increased when increased the wavelength at the concentration of 1 mM. Based on Pearson correlation, there was a significant difference between OD with an increasing wavelength of the samples. The simultaneous effect of CT, wavelength, and concentration with 95% confidence bounds have been good fitting with the first-order RPM. Therefore, the co-presence of CT, wavelength, and concentration were an interference factor on colorimetric in MTT assay especially at a wavelength = 560 nm and in amorphous nanoparticles. As the SiO_2_ concentration NPs increased, the synergistic effects of all three variables increased significantly. The best structure of the ANN using the LM algorithm was related to 3:7:1 with all datasets (0.936) and test dataset (0.934) as MSE = 0.0006. The correlation between the values predicted by the ANN model versus the actual value obtained from the laboratory data was 0.88, which indicates an acceptable correlation between the network output and the amount of OD. Sensitivity analysis using enter linear regression model was showed the beta coefficient was 0.038, − 0.429, and − 0.241 for calcination temperature of SiO_2_ NPs, nanoparticle concentration, and the wavelength of ELISA-reader, respectively. Thus, among the studied variables, the most effective factor was the concentration of nanoparticles (Supplementary Information).

Concerning our results and the effect of SiO_2_ NPs concentration and structure as well as the wavelength of MTT on OD, it is suggested to perform an MTT assay based on the control sample (i.e. the same concentration of SiO_2_ NPs in media without cell). So, the OD of control samples should decrease from case samples. Moreover, the concentration of SiO_2_ NPs should be selected based on the lowest interference effect. Although this study was investigated the effect of several parameters on the toxicity assay (MTT) for the first time, there was some limitation for this study. Therefore, it was suggested that the effect of cell media and some characteristics of SiO_2_ NPs such as transmittance and reflectance of them as optical parameters especially absorption coefficient and optical constants determine during the MTT assay. Moreover, the prediction of them was modeled by other models.

## Supplementary Information

Supplementary Information.

## Data Availability

The supporting data are available from the corresponding author on reasonable request.
