# Supplementary material for: The synergistic interference effect of silica nanoparticles concentration and the wavelength of ELISA on the colorimetric assay of cell toxicity
Source: Sci Rep. 2021 Jul 23;11:15133. doi: 10.1038/s41598-021-92419-1 (PMC8302571; doi:10.1038/s41598-021-92419-1)
Supplement: Supplementary file 1 — Supplementary Information. [file 41598_2021_92419_MOESM1_ESM.docx]

Supplementary file

Table S1. The design matrix of actual data and the best ANN model for three independent variables

|  | Temperature | Concentration | Wavelength | Actual OD | ANN predicted |  |
| --- | --- | --- | --- | --- | --- | --- |
| 1 | 70 | 1 | 470 | 0.268444 | 0.28047 |  |
| 2 | 70 | 1 | 490 | 0.350222 | 0.35466 |  |
| 3 | 70 | 1 | 520 | 0.464444 | 0.46401 |  |
| 4 | **70** | **1** | **570** | 0.504778 | **0.49074** |  |
| 5 | 70 | 10 | 470 | 0.244667 | 0.25026 |  |
| 6 | 70 | 10 | 490 | 0.307333 | 0.30327 |  |
| 7 | 70 | 10 | 520 | 0.398333 | 0.41943 |  |
| 8 | 70 | 10 | 570 | 0.422 | 0.45794 |  |
| 9 | 70 | 100 | 470 | 0.286 | 0.28835 |  |
| 10 | 70 | 100 | 490 | 0.35 | 0.33969 |  |
| 11 | 70 | 100 | 520 | 0.392222 | 0.39825 |  |
| 12 | 70 | 100 | 570 | 0.381667 | 0.37607 |  |
| 13 | 350 | 1 | 470 | 0.292556 | 0.27125 |  |
| 14 | 350 | 1 | 490 | 0.384667 | 0.35897 |  |
| 15 | 350 | 1 | 520 | 0.509889 | 0.45503 |  |
| 16 | **350** | **1** | **570** | **0.545889** | 0.48152 |  |
| 17 | 350 | 10 | 470 | 0.257778 | 0.24406 |  |
| 18 | 350 | 10 | 490 | 0.327 | 0.30866 |  |
| 19 | 350 | 10 | 520 | 0.428778 | 0.41071 |  |
| 20 | 350 | 10 | 570 | 0.453778 | 0.44513 |  |
| 21 | 350 | 100 | 470 | 0.220111 | 0.23026 |  |
| 22 | 350 | 100 | 490 | 0.258556 | 0.27493 |  |
| 23 | 350 | 100 | 520 | 0.293667 | 0.32648 |  |
| 24 | 350 | 100 | 570 | 0.285333 | 0.33642 |  |
| 25 | 600 | 1 | 470 | 0.259667 | 0.26134 |  |
| 26 | 600 | 1 | 490 | 0.343667 | 0.35874 |  |
| 27 | 600 | 1 | 520 | 0.451 | 0.44452 |  |
| 28 | 600 | 1 | 570 | 0.490556 | 0.47208 |  |
| 29 | 600 | 10 | 470 | 0.243778 | 0.23807 |  |
| 30 | 600 | 10 | 490 | 0.305111 | 0.31153 |  |
| 31 | 600 | 10 | 520 | 0.401889 | 0.40204 |  |
| 32 | 600 | 10 | 570 | 0.425333 | 0.43409 |  |
| 33 | 600 | 100 | 470 | 0.233333 | 0.21697 |  |
| 34 | 600 | 100 | 490 | 0.287222 | 0.26784 |  |
| 35 | 600 | 100 | 520 | 0.373 | 0.3286 |  |
| 36 | 600 | 100 | 570 | 0.395111 | 0.36666 |  |
| 37 | 800 | 1 | 470 | 0.272667 | 0.23956 |  |
| 38 | 800 | 1 | 490 | 0.348778 | 0.33143 |  |
| 39 | 800 | 1 | 520 | 0.461889 | 0.42049 |  |
| 40 | 800 | 1 | 570 | 0.499333 | 0.46006 |  |
| 41 | 800 | 10 | 470 | 0.212556 | 0.22339 |  |
| 42 | 800 | 10 | 490 | 0.265778 | 0.28994 |  |
| 43 | 800 | 10 | 520 | 0.356889 | 0.37782 |  |
| 44 | 800 | 10 | 570 | 0.384444 | 0.42062 |  |
| 45 | **800** | **100** | **470** | **0.192111** | **0.21173** |  |
| 46 | 800 | 100 | 490 | 0.239333 | 0.26342 |  |
| 47 | 800 | 100 | 520 | 0.314667 | 0.33923 |  |
| 48 | 800 | 100 | 570 | 0.338778 | 0.40897 |  |
| 49 | 1000 | 1 | 470 | 0.208222 | 0.228 |  |
| 50 | 1000 | 1 | 490 | 0.252556 | 0.30318 |  |
| 51 | 1000 | 1 | 520 | 0.327667 | 0.37586 |  |
| 52 | 1000 | 1 | 570 | 0.349667 | 0.42649 |  |
| 53 | 1000 | 10 | 470 | 0.247889 | 0.21623 |  |
| 54 | 1000 | 10 | 490 | 0.315111 | 0.27007 |  |
| 55 | 1000 | 10 | 520 | 0.414778 | 0.33663 |  |
| 56 | 1000 | 10 | 570 | 0.443222 | 0.3836 |  |
| 57 | 1000 | 100 | 470 | 0.218778 | 0.21925 |  |
| 58 | 1000 | 100 | 490 | 0.282111 | 0.28432 |  |
| 59 | 1000 | 100 | 520 | 0.382667 | 0.36092 |  |
| 60 | 1000 | 100 | 570 | 0.419222 | 0.41294 |  |
